# The NTP binding site of the polymerase ribozyme

**DOI:** 10.1093/nar/gky898

**Published:** 2018-10-05

**Authors:** Arvin Akoopie, Ulrich F Müller

**Affiliations:** Department of Chemistry and Biochemistry, University of California, San Diego, La Jolla, CA, USA

## Abstract

A previously developed RNA polymerase ribozyme uses nucleoside triphosphates (NTPs) to extend a primer 3′-terminus, templated by an RNA template with good fidelity, forming 3′-5′-phosphordiester bonds. Indirect evidence has suggested that the ribozyme's accessory domain binds the NTP with a highly conserved purine-rich loop. To determine the NTP binding site more precisely we evolved the ribozyme for efficient use of 6-thio guanosine triphosphate (6sGTP). 6sGTP never appeared in the evolutionary history of the ribozyme, therefore it was expected that mutations would appear at the NTP binding site, adapting to more efficient binding of 6sGTP. Indeed, the evolution identified three mutations that mediate 200-fold improved incorporation kinetics for 6sGTP. A >50-fold effect resulted from mutation A156U in the purine-rich loop, identifying the NTP binding site. This mutation acted weakly cooperative with two other beneficial mutations, C113U in the P2 stem near the catalytic site, and C79U on the surface of the catalytic domain. The preference pattern of the ribozyme for different NTPs changed when position 156 was mutated, confirming a direct contact between position 156 and the NTP. The results suggest that A156 stabilizes the NTP in the active site by a hydrogen bond to the Hoogsteen face of the NTP.

## INTRODUCTION

The RNA world hypothesis describes a stage in the early evolution of life in which RNA served as the genome and as the only genome-encoded catalyst (1–5). To test how the RNA world could have functioned, catalytic RNAs (ribozymes) are being developed that may ultimately allow establishing an RNA world organism in the lab (6). The central catalyst in an RNA world organism would be an RNA polymerase ribozyme that facilitates self-replication. A ribozyme with RNA polymerization activity was developed in 2001, the R18 polymerase ribozyme (7). Several variants of this ribozyme have been developed with improved performance (8–11) although so far, none are able to mediate self-replication.

**Figure 1.**
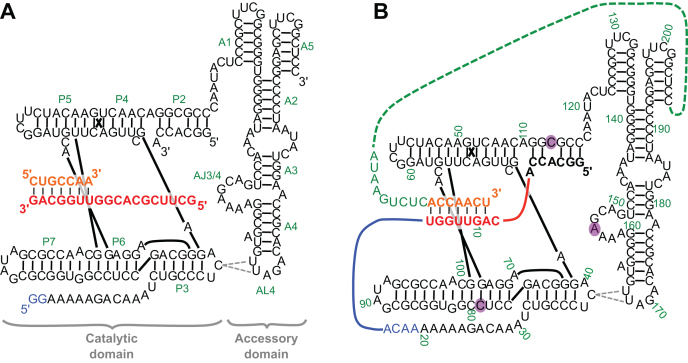
Secondary structure representations of (**A**) the R18 polymerase ribozyme (7) and (**B**) the construct used for in vitro evolution for efficient 6sGTP ligation. All nucleotides that are identical between the two constructs are shown in black. The specific secondary structure and the contact point between AL4 and C39 are based on Wang *et al*. (17). Secondary structure elements are indicated in green in (A). The secondary structure elements for the ligase core (P2-P7) are labeled consistent with Ekland *et al*. (20) and labeling for the accessory domain (A1-A5) is based on Wang *et al*. (17). The nucleotide numbering used in this study is shown in green in (B). The three nucleotides of central importance in this study (C79, C113, A156) are shown with a purple background.

The structure of the R18 polymerase ribozyme consists of two domains, the catalytic core (or ligase domain), and the accessory domain (Figure 1A). The three-dimensional structure of the catalytic core has been determined by X-ray crystallography (12,13), showing how the 3′-hydroxyl group of the extending primer is positioned for an in-line attack on the 5′-triphosphate of the incoming nucleoside triphosphate (NTP). In contrast, much less is known about the structure of the accessory domain. The accessory domain is not necessary for primer extension because three to six nucleotides can be added when a part of the catalytic core serves as template (14). However, the accessory domain becomes necessary when a primer / template duplex is bound in trans (7). This allows for longer polymerization to occur via a mostly non-processive mechanism of binding and releasing the primer / template duplex (15), held in place to a large extent by contacts to 2′-hydroxyl groups in the primer and template (16). The accessory domain is draped over the vertex of the tripod structure of the catalytic core, aided by tertiary interactions between the terminal AL4 loop of the accessory domain and the J3/4 loop within the catalytic domain (17).

The binding of NTPs appears to be mediated by a purine-rich loop in the accessory domain (17). This purine-rich loop seems to be ideally positioned near the catalytic site of the catalytic domain, and the finding that any mutation in this loop leads to a substantial drop in polymerization activity supports the idea that the purine-rich loop is the binding site for NTPs. However, these data do not generate a causal link to NTP binding, and it is still unclear how the interactions of accessory domain and catalytic core stabilize the NTP in the catalytic site.

To identify the location of the NTP binding site in a functional assay we evolved a variant of the R18 polymerase ribozyme for efficient use of 6-thio guanosine triphosphate (6sGTP). The polymerase ribozyme has never encountered this specific NTP in its evolution/design history (7,14,18–20). Therefore, evolving the polymerase ribozyme towards utilizing 6sGTP would be expected to enrich for mutations that reveal the NTP binding site. During the evolution, the mutations were allowed to occur throughout the ribozyme sequence to detect any interactions that may assist NTP binding. The most efficient isolate from the evolution had >200-fold improved 6sGTP ligation kinetics and relied on three mutations. The mutation A156U in the purine-rich loop was responsible for >50-fold improvement, suggesting a direct contact of A156 with the nucleobase of the incoming NTP. The direct interaction between A156 and the NTP was confirmed in experiments where A156 was mutated, which changed the NTP preference pattern. Mutations in position 79 and 113 had less than 3-fold effects and were weakly cooperative with A156U but not with each other. The resulting network of interactions is consistent with existing structural data and builds a picture of how the dynamic interactions in the polymerase ribozyme mediate RNA polymerization.

## MATERIALS AND METHODS

### Generation of the polymerase ribozyme construct

The polymerase ribozyme construct for the evolution was generated by PCR amplification from a plasmid containing the R18 polymerase ribozyme (7). A second PCR reaction added a T7 promoter and a hammerhead ribozyme on the 5′-end similar to previous work (21), and a linker and DNAzyme recognition site (see below) on the 3′ end. The resulting sequence of the DNA construct was 5′-*AATTTAATACGACTCACTATA*gggtggtgccctgacgagctaagcgaaactgcggaaacgcagtcGGCACCACAGTTGGTACAAAAAAAGACAAATCTGCCCTCAGAGCTTGAGAACATCTTCGGATGCAGAGGAGGCAGCCTCCGGTGGCGCGATAGCGCCAACGTTCTCAACAGGCGCCCAATACTCCCGCTTCGGCGGGTGGGGATAACACCTGACGAAAAGGCGATGTTAGACACGCCAAGGTCATAATCCCCGGAGCTTCGGCTCCNNNNNNNNNNNNNNNATAAGTCTCACCAACT/TATATGTTCTAGCGCGGA-3′, where the italicized letters indicate the T7 promoter, the lowercase letters indicate the hammerhead ribozyme, the underlined regions indicate primer binding sites for PCR and reverse transcription, the N_15_ sequence is the linker sequence (see two paragraphs further down), and the sequence downstream of the linker sequence shows the binding site of the DNAzyme with the cleavage site marked as ‘/’ (see next paragraph). The RNA sequence was generated by in vitro run-off transcription from this PCR product with T7 RNA polymerase under standard conditions, and purification by denaturing PAGE.

### Processing of ribozyme 3′-ends with a DNAzyme

To generate a homogeneous 3′-terminus of the purified RNA transcript with 2′- and 3′-hydroxyl groups, a recently developed DNAzyme was employed that generates a 5′-phosphate and 2′,3′-hydroxyl groups (22). To do this the DNAzyme variant 9SK17 was modified for better complementary to the pool 3′-terminus, resulting in the sequence 5′-GCGCTAGAACATGCCAGCGATCAAAGACGGCGAGTTGTACCCATAGGTGTCTAGTtggTGAGACTT-3′ where the underlined sequences were complementary to the substrate, and the three lowercase nucleotides differ from 9SK17. The pool transcript was heat renatured (3 min/90°C) with a 2-fold stoichiometric excess of the DNAzyme, chilled on ice for 10 min, and incubated at 37°C for 4 h with the final concentrations of 70 mM HEPES/NaOH pH 7.5, 150 mM NaCl, 1 mM ZnCl_2_, 2 mM HNO_3_, 20 mM MnCl_2_, and 40 mM MgCl_2_. The ZnCl_2_ stock solution was a mixture of 10 mM ZnCl_2_ with 20 mM HNO_3_ and 200 mM HEPES/NaOH pH 7.5. The reaction was stopped by adding an excess of Na_2_EDTA over divalent cations, and adding formamide to a final concentration of 50% (v/v). The processed RNA pool was purified by denaturing 5% polyacrylamide gel electrophoresis, resulting in a yield of ∼50% for the processed pool RNA.

### Development of linker sequences between ribozyme 3′-terminus and primer 5′-terminus

To allow self-tagging of efficient polymerase ribozymes the pool 3′-terminus was covalently linked to the primer 5′-terminus (see Figure 1B). The crystal structure of the ligase ribozyme (13) suggested that a linker length of 15 nucleotides would be sufficient for this link. To identify linker sequences that would allow the ribozyme to be active, 15 randomized nucleotides were positioned between the ribozyme 3′-terminus and the primer 5′-terminus (see’ Generation of the polymerase ribozyme construct). The goal was not to identify the best linker sequence because such a sequence might have fine-tuned, and therefore modified the ribozyme conformation. Instead, three sequences were arbitrarily chosen from a large number of linker sequences that permitted ligation. To identify such linker sequences, one round of in vitro selection with 6sGTP was carried out. For this selection, a total amount of 3 pmol of the pool with the randomized linker sequence was heat-denatured in the presence of 50 mM Tris / HCl pH 8.3 at 80°C for 2 min followed by immediate chilling on ice for five minutes. The ligation was started by adding a premix such that the final concentration of all the components were 100 mM MgCl_2_, 200 mM KCl, 1% PEG 20,000, 50 mM Tris/HCl pH 8.3, and 20 μM 6-thio GTP (Axxora). The mixture was incubated at 22°C for 3 h. The RNA was ethanol precipitated and separated by APM PAGE as described (23–25), with a total concentration of 240 μM immobilized aminophenyl-mercury in the APM layer. Note that the 10× stock solution was 2.4 mM APM in DMSO. The material at the APM interface was excised, eluted with 300 mM NaCl and 5 mM DTT, ethanol precipitated, reverse transcribed using Superscript III (NEB), PCR amplified, cloned into pUC19 plasmids, and sequenced. A total of seven individual clones were tested for their ability to generate a signal at the APM interface when the procedure of the selection step was performed with individual ribozyme constructs that were internally labelled with α[^32^P]-ATP. The three best performing sequences were chosen for the following in vitro evolution (see below).

### Evolution of ribozyme constructs with improved 6sGTP ligation efficiency

The polymerase ribozyme construct as shown under ‘generation of the polymerase ribozyme construct’ was subjected to 10 rounds of *in vitro* evolution for more efficient use of 6sGTP as substrate for ligation to its own 3′-terminus. In the first round of evolution, sequence diversity in the polymerase ribozyme sequence was generated by 30 cycles of mutagenic PCR (26). The mutagenesis rate was about 1.0 mutations per 10 cycles of mutagenic PCR and per 100 mutagenized positions (27), consistent with the original publication of the procedure (28). We previously found a bias among the six possible mutations with 23% AT→GC, 38% AT→TA, 18% GC→AT, 11% GC→TA, 6% AT→CG and 5% GC→CG (27). Of the 206 nucleotides in the polymerase ribozyme construct, 17 nucleotides on each end of the construct were fixed and not mutated during the evolution to allow primer binding for reverse transcription and PCR. In the following rounds of evolution, no mutagenic PCR was employed with exception of mutations generated by the intrinsic mutagenicity of Taq DNA polymerase.

During transcription of the pool RNA, the 5′-terminal hammerhead ribozyme removed itself, generating a 5′-hydroxyl group as described earlier (21). Transcribed pool RNAs were purified via 5% PAGE and processed with the DNAzyme as described two sections above. During transcription, the pool RNA molecules were internally labeled by incorporating trace amounts of α-[^32^P] ATP. This allowed locating the molecules at the APM interface for excision, as well as monitoring the fraction of pool reacting during the selective step, over the course of the evolution.

The incubation of the pool with 6sGTP was performed under the same conditions as used in the linker selection (see the previous section). The linker sequences in the ribozyme construct were alternated during the evolution to prevent the polymerase ribozyme from evolving specific sequences that interact with these linkers, with 5′-CGCCUAGACCCACGC-3′ (rounds 1, 4, 7, 10), 5′-GCUCACACAAGAAAA-3′ (rounds 2, 5, 8) and 5′-CAGAACUCCAAUAUA-3′ (rounds 3, 6, 9). The concentration of 6-thio GTP, and the incubation time were decreased over the rounds of evolution to increase selection stringency (see Supplementary Figure S1). After reverse transcription of the selected RNAs, a first PCR reaction amplified the selected sequences with short PCR primers corresponding to the underlined sequence in section ‘generation of the polymerase ribozyme construct’. A second PCR reaction used PCR primers with long 5′-extensions to re-generate the pool 5′-terminus with the promoter for T7 RNA polymerase, hammerhead ribozyme, and the pool 3′-terminus with linker sequence and DNAzyme target site. The resulting PCR product was the input for the next round of evolution.

After evolution round ten, the PCR products were appended with restriction sites, cloned into the plasmid pUC19, and sequenced. To test the activity of individual clones, the sequence of each clone was amplified as described in the first two paragraphs of this section.

### Ligation assay

**Figure 2.**
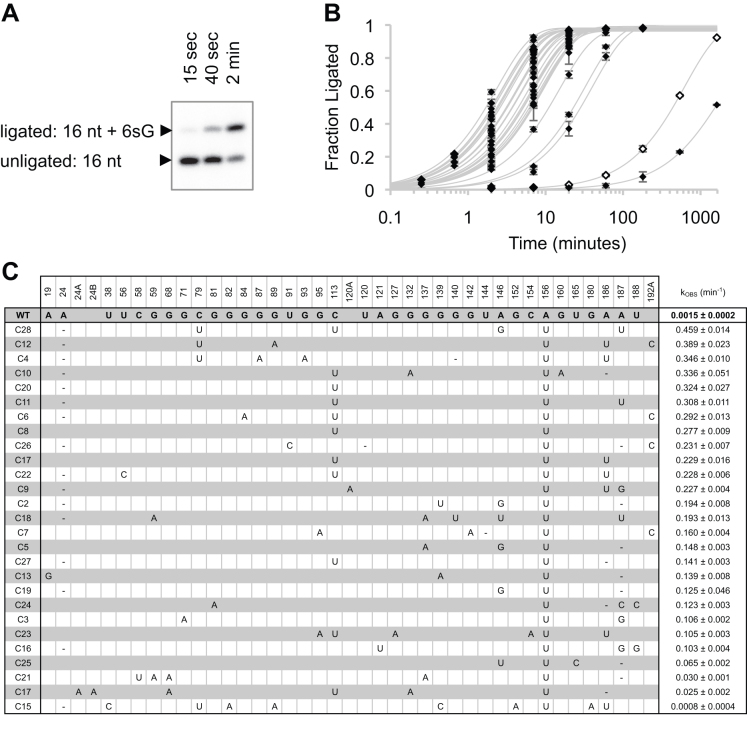
Analysis of 27 arbitrarily chosen isolates from round ten of the *in vitro* evolution. (**A**) Representative phosphorimage of PAGE-separated ligation products of the most active clone 28 with 25 μM 6sGTP. This reaction did not use a linker between the primer 5′-terminus and the ribozyme 3′-terminus, therefore the primer was elongated from 16 nt to 17 nt. The reaction times as well as the identity of the signals are indicated. (**B**) Ligation kinetics of the 27 ribozymes at 25 μM 6sGTP. Time points were taken at 2, 7, 20, 60 and 180 min. Additional samples were taken for weakly active clones (WT and C15) at 9 h and 27 h, and for highly active clones at 15 and 40 seconds. Error bars are standard deviations from triplicate experiments. (**C**) Correlation between ribozyme mutations and their 6sGTP ligation kinetics. All positions that show at least one mutation in the 27 isolates are given on the top. The clone name is given on the left. Mutations relative to the wild type (WT) sequence of the R18 ribozyme are given in the central part. For WT the unmutated nucleotide is shown, while all other clones show only the mutated nucleotide. An empty space in the WT row with nucleotides for the isolates indicates insertion events in at least one of the selected sequences. The clones are sorted according to their ligation kinetics, with values and their standard deviations given in the right column. Note that the values of these rates are slight overestimates due to the reaction time points used in this screen.

**Figure 3.**
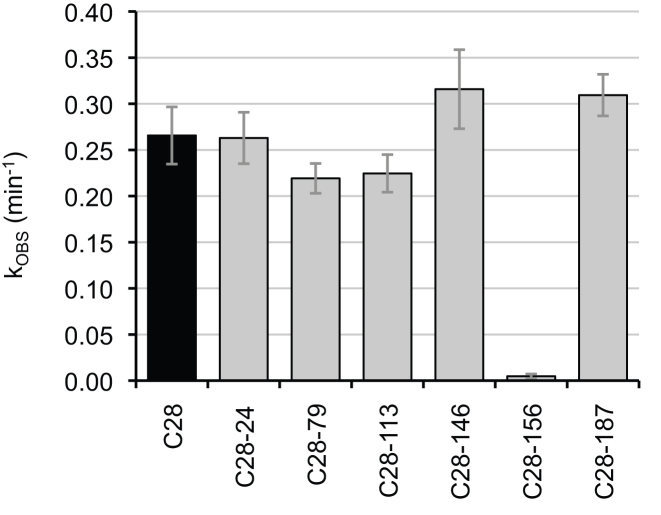
Analysis of the most efficient isolate of the evolution, clone 28. Shown are the effects on the ligation rate, by individually removing each of its six mutations. The name of each polymerase ribozyme variant is given below, where C28 indicates all six mutations and the additional number indicates which of the six mutations was removed. The observed rates were obtained by single-exponential fitting to reaction time courses. Removing mutations C79U or C113U resulted in a 1.2-fold reduction, and removing mutation A156U resulted in a 56-fold reduction. Error bars are standard deviations from three independent experiments.

**Figure 4.**
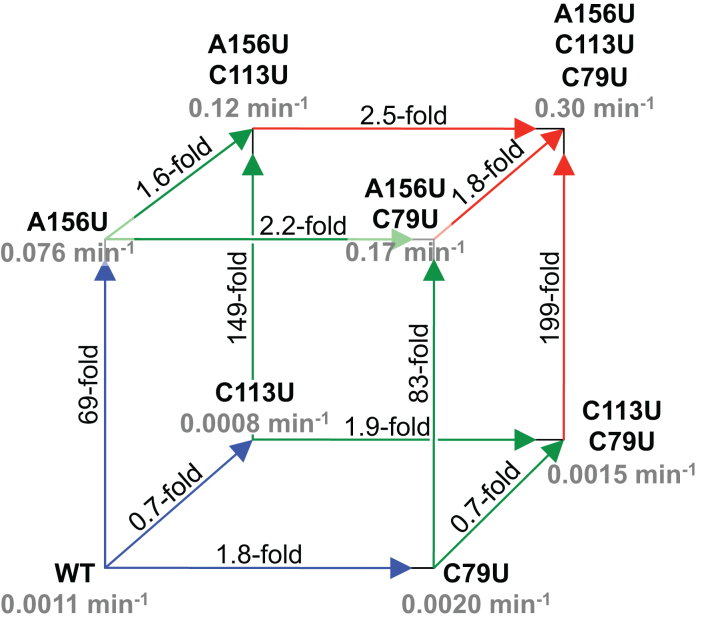
Analysis of cooperative effects between the three beneficial mutations. The ‘kinetic cube’ shows all intermediates from the wild-type (WT) to the triple mutant (A156U, C113U, C79U), with the 6sGTP ligation rate at 25 μM 6sGTP indicated in gray. The paths of the first mutation from the WT are labeled in blue, of the second mutation in green, and of the third mutation in red. For each path the -fold increase in ligation kinetics is indicated. The paths *up* correspond to A156U mutations, the paths *back* to the C113U mutation, and the paths *right* to C79U mutations. The comparison of the four different effects for each mutation showed whether mutations acted cooperatively (see text).

To measure the ligation rate for individual ribozyme sequences they were transcribed, processed at their 3′-termini, and purified as described above. The RNA substrate 5′-AUAAGUCUCACCAACU-3′ was transcribed with a 5′-terminal hammerhead ribozyme, which removed itself during transcription as described before (21). After 5′-radiolabeling with polynucleotide kinase, trace amounts of the radiolabeled substrate were dissolved with the ribozymes at 1.5 μM concentration in 500 mM Tris/HCl pH 8.3. After heat renaturing for two minutes at 80°C the samples were immediately transferred to ice for five minutes. The mixture was then diluted to a final concentration of 100 nM ribozyme, 50 mM Tris/HCl pH 8.3, freshly prepared 1% (w/v) PEG 20 000, 100 mM MgCl_2_, 200 mM KCl and 25 μM 6sGTP. At given time points 5 μl aliquots of the reaction were quenched with 5 μL formamide gel loading buffer containing an excess of Na_2_EDTA over Mg^2+^ from the reaction. Samples were separated by denaturing 20% PAGE, exposed to phosphorimager screens, then scanned and quantitated on a Typhoon phosphorimager (GE) using the Quantity One software (Bio-Rad). Rate constants were obtained by single-exponential curve fitting to the data in Microsoft Excel, using the solver add-on. For data in Figures 2–4, the amplitude for fitted curves was set to 100% because all constructs with fast ligation kinetics approached 100% in saturation. For data in Figure 6, the amplitude was set to 80% because all constructs with fast kinetics approached 80% in saturation. The difference between these amplitudes appeared to be in the preparation of the substrates. Ligation rates for canonical NTPs were measured exactly the same, only by substituting 6sGTP with the respective NTP.

The k_OBS_ for C28 was higher in the initial screen than in the side-by-side comparisons with its variants because in the initial screen included very short timepoints (15 s, 45 s) for the fastest variants. In contrast, the analysis of the variants of C28 started at 2 min. Because the kinetics of clone 28 deviated somewhat from first order kinetics the inclusion or omission of the first two timepoints led to slightly different values for *k*_OBS_.

## RESULTS

To identify the NTP binding site of the polymerase ribozyme, the ribozyme was evolved for efficient use of 6-thio guanosine triphosphate (6sGTP) as substrate instead of canonical NTPs. The rationale was that the polymerase ribozyme had never encountered 6sGTP in its evolutionary history. Therefore, evolutionary optimization of the ribozyme for efficient use of 6sGTP was expected to lead to beneficial mutations especially at those nucleotides that were responsible for binding NTPs.

The construct for the polymerase ribozyme evolution was generated such that successful polymerase ribozymes would tag their own 3′-terminus with 6sGTP (Figure 1B). To do this, the R18 polymerase ribozyme was modified in two ways. First, the primer binding mode was converted to the format of its evolutionary ancestor, the class I ligase ribozyme (20) such that the primer's 3′-terminus was paired near the ribozyme's active site, using the neighboring C nucleotide as templating base. This primer binding mode is structurally equivalent to the trans binding mode of the polymerase ribozyme, based on experiments tethering the primer-template duplex to different positions of the polymerase ribozyme (17). Second, the 3′-terminus of the polymerase ribozyme was linked to the 5′-terminus of the primer. Three different linker sequences were identified by a single low-stringency selection step from a pool of linkers with 15 randomized nucleotides (see materials and methods). To generate 3′-hydroxyl termini for the new, elongated ribozyme constructs, transcripts were processed using a catalytic DNA (DNAzyme) that site-specifically cleaves RNA, leaving the upstream cleavage product with a 3′-hydroxyl group (22). In this ribozyme construct, a 3′-elongation of the primer with 6sGTP would tag the 3′-terminus of the ribozyme with a sulfur-containing nucleotide. This sulfur tag was used to separate active from inactive polymerase ribozyme variants by denaturing polyacrylamide gel electrophoresis with covalently immobilized aminophenyl mercury (APM-PAGE) (23–25). This setup allowed the selection of active ribozymes from pools of ribozyme variants.

The starting pool for the evolution was prepared by mutagenic PCR, introducing mutations over the entire length of the polymerase ribozyme (26,28). Subsequent rounds of evolution did not use explicitly mutagenic conditions for PCR. However, Taq polymerase was used for PCR amplification, which introduced additional mutations via its inherent error rate. The stringency of the selection step was successively increased over ten evolution rounds, from a 6sGTP concentration of 20 μM and an incubation time of 3 h to a 6sGTP concentration of 0.5 μM and an incubation time of 15 minutes (Supplementary Figure S1). The fraction of pool selected at the interface of the APM gel suggested that pool activity increased 86-fold from evolution round one to ten.

To identify the most efficient ribozyme sequence for 6sGTP ligation, 27 clones from evolution round ten were tested for ligation activity with 6sGTP (Figure 2A, B). Sorting the 27 sequences according to their activity showed that a small number of mutations were highly enriched in the most efficient clones, specifically mutations A156U, C113U and C79U (Figure 2C). The enrichment of mutations in the most active clones suggested that mutation A156U had the strongest effect on 6sGTP ligation, and C79U and C113U also contributed to high activity. The most efficient isolate was clone 28, which contained six mutations compared to the parent ribozyme that included all three enriched mutations A156U, C79U and C113U.

To identify the mutations that were necessary for the high activity of clone 28 the six mutations were individually removed (Figure 3). The most dramatic effect came from mutation A156U, which dropped the activity of clone 28 by 92-fold when deleted. Two additional mutations appeared to be beneficial, mutations C79U and C113U, which each dropped activity of clone 28 by ∼1.3-fold when deleted. Mutation A24Δ did not appear to benefit 6sGTP ligation activity, and mutations A146G and A187U appeared to have none or a mildly deleterious effect on ligation kinetics. This showed that in the context of the other mutations in clone 28, only the three mutations A156U, C79U, and C113U were necessary for full activity. To test whether the three mutations A156U, C113U, and C79U alone were sufficient for full activity of clone 28 they were inserted into the wild-type sequence. The triple-mutant showed the same 6sGTP ligation kinetics as clone 28, within error, showing that A156U, C113U, and C79U were sufficient for a >200-fold improvement in the kinetics of 6sGTP ligation. Because these data were recorded at a 6sGTP concentration of 25 μM, the *k*_OBS_ = 0.30 min^−1^ corresponds to a first-order rate constant of 200 M^−1^ s^−1^.

To test the contribution of each mutation in the triple mutant, all intermediates between the wild-type sequence and the triple mutant were constructed, and their 6sGTP ligation kinetics were measured (Figure 4). The beneficial effect of mutation A156U was 69-fold to 199-fold, depending on the mutational background, and effects were <3-fold for mutations C113U and C79U. The simplest explanation for the strong effect of A156U is that the nucleobase of A156 in the purine-rich loop is directly involved in NTP recognition.

The mutations A156U and C113U acted weakly cooperative, as did the mutations A156U and C79U (Figure 4). In the absence of C113U, mutation A156U increased activity 69-fold and 83-fold, while in the presence of C113U, A156 increased activity 149-fold and 199-fold. This is a 2.2-fold and 2.4-fold cooperative effect, respectively. Similarly, mutation C113U increased the 6sGTP ligation rate 1.6-fold and 1.8-fold in the presence of A156U, while decreasing the rate 1.3-fold and 1.4-fold in the absence of A156U, confirming the cooperative effect between these two mutations. Mutation A156U also acted weakly cooperative with mutation C79U: The beneficial effects of mutation A156U in the presence of C79U (83-fold and 199-fold) are 1.2-fold and 1.3-fold stronger than in the absence of C79U (69-fold and 149-fold). Similarly, the beneficial effects of C79U in the presence of A156U (2.2-fold and 2.5-fold) were 1.2-fold and 1.9-fold stronger than in the absence of C79U. There was no cooperative effect between mutations C113U and C79U, with influences of 1.0-fold and 1.1-fold. Together, these results suggested a network of direct or indirect interactions between the three nucleotides: A156 contacts the incoming NTP, and A156 interacts directly or indirectly with C113 in the P2 duplex, and with C79 in the body of the ligase core. The nucleobases of C79 and C113 did not seem to interact.

**Figure 5.**
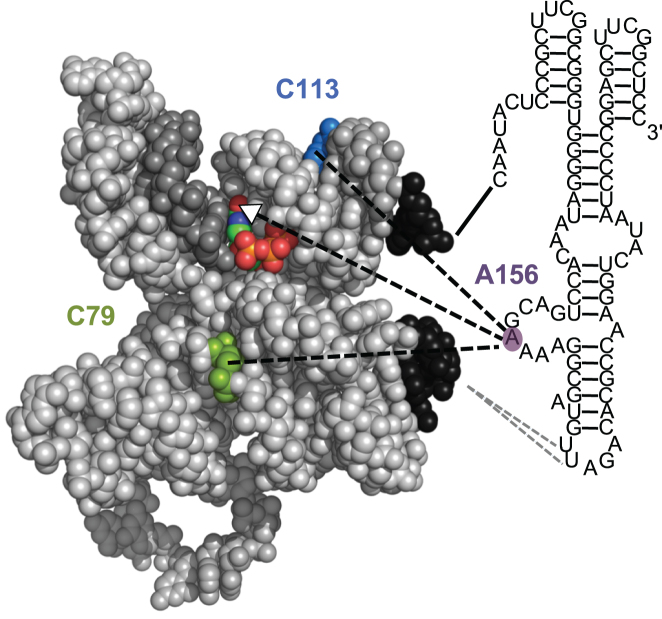
Model for the interaction between A156 (purple background) and the ligase core (gray). The NTP in the active site (atom colors), position C79 (green), and position C113 (blue) are highlighted. The results of this study suggest a direct interaction between A156 and the NTP, and indirect interactions (dashed lines) between A156 and C79, and between A156 and C113. The white arrowhead of the dashed line between A156 and the NTP points towards position 6 of the NTP nucleobase, which may serve as hydrogen donor to position 156 (see text). Note that the X-ray structure corresponds to GTP in the active site therefore the red sphere (oxygen) corresponds to the 6-thio position of 6sGTP.

The simplest structural explanation for these cooperativity data is that A156 directly interacts with the incoming NTP, and that C79 as well as C113 interact with A156 but not with each other (Figure 5). The direct interaction between A156 and the incoming NTP must be with the Hoogsteen face of the incoming NTP because only this side of the NTP is accessible on the surface of the catalytic core (13). The nucleobase of A156 would have to be positioned into a narrow cleft, perhaps benefitting from stacking interactions with the P2 helix.

**Figure 6.**
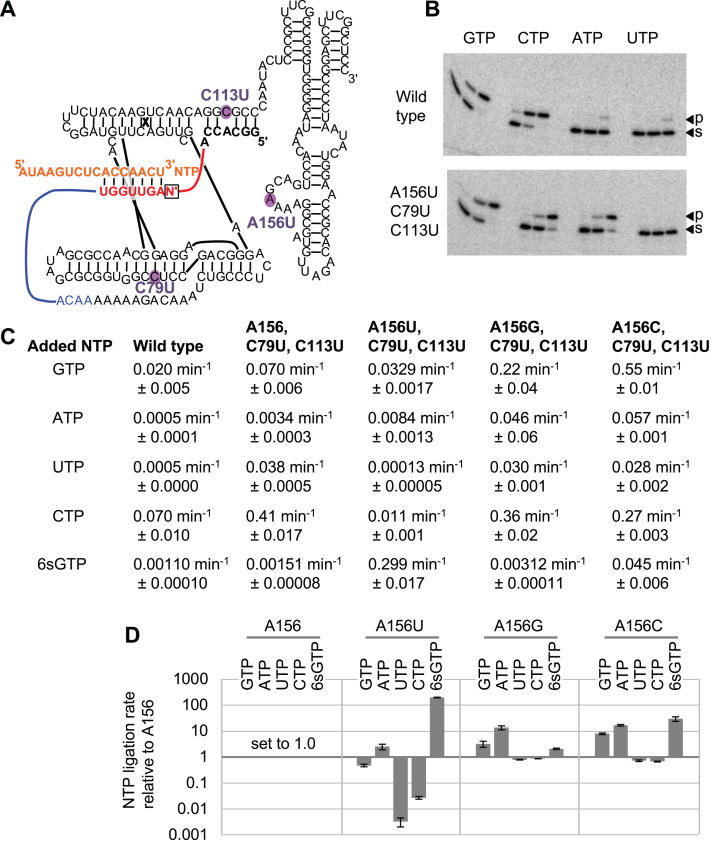
Influence of mutations in the polymerase ribozyme on the ligation kinetics of canonical NTPs and 6sGTP. (**A**) Secondary structure representation of the polymerase ribozyme constructs used for this assay. The three positions differing between wild type and triple mutant are highlighted in purple, with a purple label showing the mutation. The primer (orange) is base paired to the template strand (red) and is extended by the incoming NTP (red). In each experiment, the templating nucleotide (boxed) is adjusted to form a Watson–Crick pair with the NTP. The sequence linking the R18 polymerase ribozyme 5′-terminus with the template 3′-terminus is shown in blue, and the red line shows the connection between template 5′-terminus and the 3′-terminus of the 5′-GGCACCA sequence in the P2 stem. (**B**) Autoradiogram of radiolabeled ligation products that were separated by denaturing 10% PAGE. The NTPs used for each reaction are shown on the top. The three lanes for each NTP stem from samples with the incubation times of 2, 20 and 180 min. The lower band (labeled with ‘s’ on the right) is the substrate, the upper band (p) is the product, which is one nucleotide longer than the substrate. The upper and lower audioradiogram show the signals from the wild type sequence and the triple mutant A156U/C79U/C113U, respectively. (**C**) Observed kinetic rates determined from triplicate experiments as shown in B, using single-exponential curve fitting to quantified data. (**D**) NTP ligation rates relative to the variant A156/C79U/C113U. All variants contain the C79U and C113U mutation, and only the nucleotide in position 156 is varied. The NTP used for each experiment is given above each column. Error bars correspond to standard deviations from triplicate experiments.

If there is a direct contact between position 156 and the incoming NTP then mutations in position 156 should modulate the preference for NTPs. To test this, the NTP ligation rates were measured for different polymerase ribozyme constructs (Figure 6). In the background of mutations C79U and C113U, the NTP ligation rates were compared to the construct with the wild-type A in position 156 (Figure 6D). While the mutant containing A156G showed a similar NTP preference as A156C, there were strong differences between A156, A156U and A156G/C. If position 156 would act via influencing catalysis then the influence on NTP ligation kinetics would be expected to be similar between the NTPs. Therefore, the different NTP preference patterns caused by mutations in position 156 confirm that position 156 is directly involved in binding the incoming NTP.

The crystal structure of the ligase core confines the possible contacts of the purine-rich loop to the Hoogsteen face of the NTP (13) because the Watson-Crick face is involved in base pairing to the templating nucleotide, and the minor groove is inaccessible due to the position of the accessory domain (Figure 5). The Hoogsteen face of 6sGTP displays the thio-modification, which largely populates the thiol form under the used reaction conditions ((29) and Supplementary Figure S2). The 6-thiol may act as a hydrogen donor similar to the exocyclic amino group of adenine, which would explain why the mutation A156U prefers both 6sGTP and ATP but discriminates against GTP, UTP, and CTP (Figure 6D). We did not identify a similarly simple explanation for the NTP preference of mutations A156G and A156C.

## DISCUSSION

The results of this study showed that mutation A156U had a >50-fold, beneficial effect on binding of 6sGTP, and that mutations C79U and C113U, with less than 3-fold beneficial effects, acted weakly cooperative with A156U but not with each other. A direct interaction between NTP and position 156 was confirmed by changes in the NTP preference patterns due to mutations in position 156. The three proposed interactions (A156-NTP, A156-C79, and A156-C113) are consistent with the crystal structure of the ligase core, crosslinking data between loop AL4 of the accessory domain and J3/4 of the ligase domain, and mutational data of the AJ3/4 loop (17). In this bigger picture, the accessory domain is draped over the tip of the ‘tripod structure’ of the ligase core, with the purine-rich loop AJ3/4 of the accessory domain positioned to help stabilize an NTP in the active site (17). The accessory domain does not form a straight connection between its two contact points to the catalytic core (black sequences in Figure 5) but rather needs to curve to the NTP binding site. This curvature is consistent with the requirement for the bulged A164, which distorts the A4 helix (Figure 1A) to correctly dock with the ligase core (17).

The weak cooperativity between mutations A156U and C113U is consistent with A156U being in physical contact with the incoming NTP because C113 resides in the P2 helix, adjacent to the catalytic site (Figure 5). Because C113 is positioned on the opposite side of the P2 duplex as the incoming NTP (and therefore A156) we suggest that the cooperative effect between A156U and C113U results from the stabilization of the A-form duplex by the C113U mutation, by converting a C:A pair to a U:A pair. In contrast, a C:A mispair in the P2 helix makes the polymerization of multiple nucleotides more efficient (30), suggesting that a deformation in the A-form of the P2 helix is necessary for renewed binding of NTPs, or for release and re-binding of the primer/template duplex (15). The weak cooperative effect between A156U and C79U is consistent with the purine-rich loop positioning the NTP into the active site because C79 is close to the NTP in the catalytic site: The distance of 12 Å between the 2′-oxygen of C79 and a non-bridging oxygen in the α-phosphate of the NTP (13) could easily be spanned by one or two nucleotides of the eight-nucleotide purine-rich loop.

Two of the constructs tested in Figure 6 show absolute rates of NTP ligation that are higher for all canonical NTPs when compared to the wild type (‘round 18’) polymerase ribozyme. Variant A156/C79U/C113U and variant A156G/C79U/C113U show higher rates for the addition of all canonical NTPs. In this case the major benefit comes from mutations C79U and C113U because no mutation is necessary at position 156. The mutation C79U was identified earlier, as one of four mutations that improved the efficiency of the polymerase ribozyme (9). Note that mutation C113U is linked to the context of ligating single NTPs because C113U removes a mismatch in the P2 helix, which harms the function of the polymerase ribozyme (30).

To test whether the three evolved mutations were beneficial in the context of RNA polymerization in trans, the R18 polymerase ribozyme (7) was compared to the identical construct with the three mutations C79U, C113U and A156U in a polymerization assay. The results showed no detectable polymerization products for the triple mutant (data not shown). This was expected because the tested template selected for UTP as the first incoming NTP, which was bound much weaker by the triple mutant than by the wild-type sequence in the context of NTP ligation. Together, these data confirmed that the mutation A156U was a specific adaptation for 6sGTP binding.

This study showed that the sequence of the purine-rich loop has a dramatic effect on the efficiency of the polymerase ribozyme, via NTP binding. It may now be possible to develop more efficient variants of the polymerase ribozyme by replacing the purine-rich loop with a completely randomized sequence and re-selecting from this pool. The small size of this loop would allow complete coverage of all sequence variants so that loops with different sizes could be explored in the same experiment. Similarly, the results in this study can improve the use of the polymerase ribozyme as tool. A very recent study showed that the polymerase ribozyme can be used for 3′-tagging of RNA transcripts with chemically modified NTPs (31). By re-selecting the most efficient purine-rich loop sequences for 3′-tagging with chemically modified NTPs, variants of the polymerase ribozyme would emerge for efficient 3′-tagging with different chemical modifications. The only difference from the selection system in this study would be that the selection step would have to be adjusted for each chemically modified NTP: While thio-modified NTPs could be selected with the same APM-PAGEs used in this study, biotinylated NTPs could be captured by streptavidine beads, and azido modified NTPs could use click chemistry for the selection step.

## Supplementary Material

Supplementary Data
