# Supplementary material for: The NTP binding site of the polymerase ribozyme
Source: Nucleic Acids Res. 2018 Oct 5;46(20):10589–97. doi: 10.1093/nar/gky898 (PMC6237761; doi:10.1093/nar/gky898)
Supplement: Supplementary Data [file gky898_supplemental_files.pdf]

Figure S1

A

	[6 thio GTP]	Reaction time	Percent of pool at the APM interface
Round 1*	20 uM	180 min	10 %
Round 2	20 uM	180 min	8 %
Round 3	20 uM	180 min	8 %
Round 4	10 uM	180 min	15 %
Round 5	5 uM	180 min	3.7 %
Round 6	2.5 uM	180 min	19 %
Round 7	1 uM	180 min	5 %
Round 8	0.5 uM	180 min	22 %
Round 9	0.5 uM	30 min	2.5 %
Round 10	0.5 uM	15 min	1.8 %

*included mutagenic PCR

B

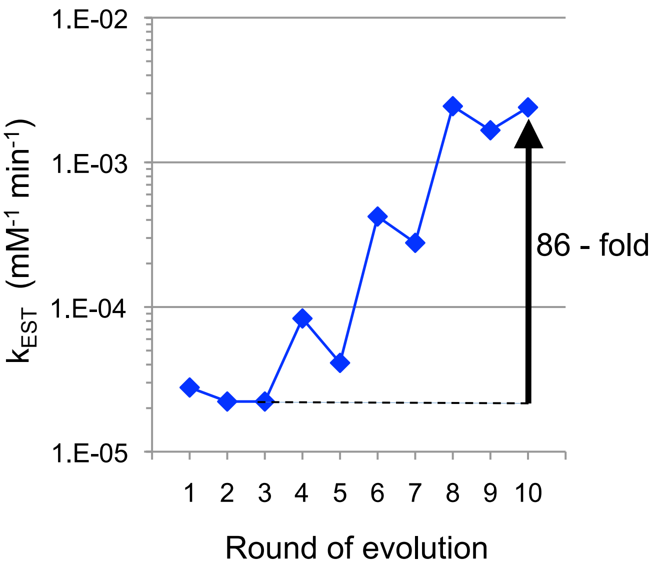

Figure S1. Progress of the in vitro evolution of the R18 polymerase ribozyme construct for the efficient ligation of 6sGTP. **(A)** This table lists the conditions for each round of the evolution. The micromolar concentration of 6-thio-GTP (6sGTP) is listed together with the reaction time between ribozyme construct and 6sGTP. The percent of pool that could be quantified at the APM interface, and that constituted the selected population during each selection step is shown on the right. **(B)** Estimated average ligation rate of the pool, as a function of the rounds of evolution. To calculate this rate, the fraction of ligated pool was divided by the concentration of 6sGTP (in miilimolar) and by the reaction time (in minutes). The results suggested that the average pool 6sGTP ligation rate increased 86-fold over the course of the evolution experiment.

Figure S2

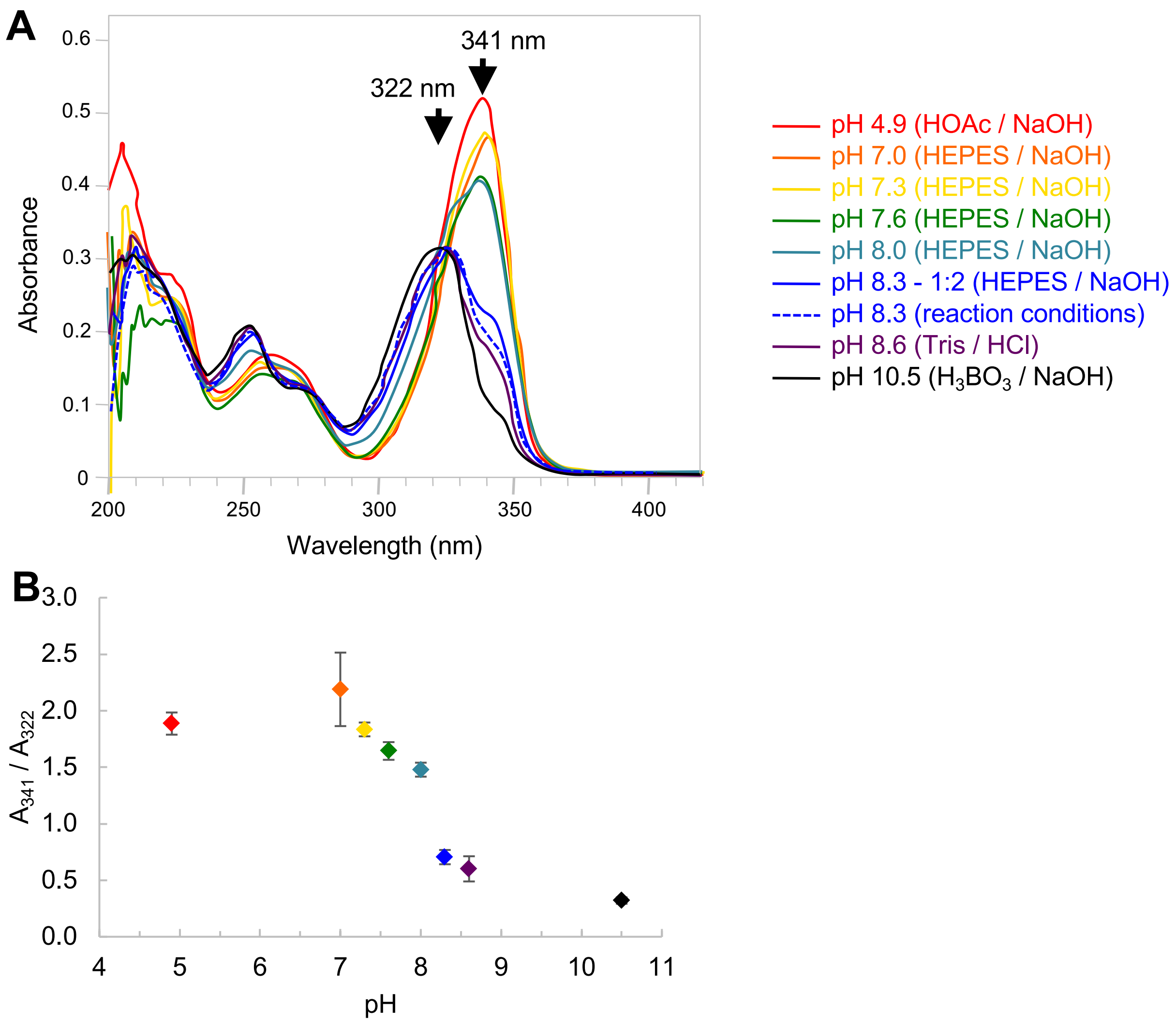

Figure S2. The thiol tautomer of 6-thio guanosine is populated at ribozyme reaction conditions. **(A)** UV absorption spectra of 1 mM 6-thio guanosine as function of pH, in 100 mM aqueous buffer. The pH and the buffer type are given on the right, in color code. The curves for each buffer are shown in the graph in the respective color. The spectrum with the dashed, blue line was recorded at ribozyme reaction conditions (50 mM Tris/HCl pH 8.3, 100 mM $MgCl_2$, 200 mM KCl, 1% (w/v) PEG 20,000). The spectra were recorded with a path length of 0.2 mm in a Nanophotometer (Implen). The peaks at 341 nm (indicating the thione form) and at 322 nm (indicating the thiolate form) are indicated with arrowheads. At ribozyme reaction conditions (dashed, blue curve) the majority of the molecules populate the thiolate form. All spectra are representatives of triplicate experiments. **(B)** Plot of the ratio of absorbances at 341 nm and at 322 nm, as a function of pH. The symbols are colored to correlate with the respective curve in (A). The strongest shift in the ratio occurs between pH 8.0 (turquoise) and 8.3 (blue). At ribozyme reaction conditions (pH ~8.3) the majority of the molecules populate the thiolate form. Error bars are standard deviations from triplicatre experiments. When error bars are not visible they are smaller than the symbol.
